# Phycobiliproteins from microalgae: research progress in sustainable production and extraction processes

**DOI:** 10.1186/s13068-023-02387-z

**Published:** 2023-11-08

**Authors:** Jinxin Wang, Song Qin, Jian Lin, Qi Wang, Wenjun Li, Yonglin Gao

**Affiliations:** 1 College of Life Sciences, Yantai University, Yantai, 264005 China; 2 Yantai Institute of Coastal Zone Research, Chinese Academy of Sciences, Yantai, 264003 China; 3 Shandong University of Traditional Chinese Medicine, Ji’nan, 250355 China

**Keywords:** Phycobiliproteins, Algae culture, Cell disruption, Separation and purification

## Abstract

Phycobiliproteins (PBPs), one of the functional proteins from algae, are natural pigment–protein complex containing various amino acids and phycobilins. It has various activities, such as anti-inflammatory and antioxidant properties. And are potential for applications in food, cosmetics, and biomedicine. Improving their metabolic yield is of great interest. Microalgaes are one of the important sources of PBPs, with high growth rate and have the potential for large-scale production. The key to large-scale PBPs production depends on accumulation and recovery of massive productive alga in the upstream stage and the efficiency of microalgae cells breakup and extract PBPs in the downstream stage. Therefore, we reviewed the status quo in the research and development of PBPs production, summarized the advances in each stage and the feasibility of scaled-up production, and demonstrated challenges and future directions in this field**.**

## Introduction

Phycobiliproteins (PBPs) are pigment protein naturally produced by cyanobacteria, red algae, and some cryptophytes. This pigment complex is a key member of photosynthesis in algae and act as a photosynthetic light-harvester, with which the efficiency of light capture in visible spectrum can be enhanced [1]. PBPs consists of deacylated protein and phycobilins bound by covalent bonds [2], and can be classified specifically into four categories in spectral property, namely, phycoerythrin (PE; λ_max_ = 490–570 nm), phycocyanin (PC; λ_max_ = 610–625 nm), allophycocyanin (APC; λ_max_ = 650–660 nm), and phycoerythrocyanin (PEC; λ_max_ = 560–600 nm) [3].

As a type of natural pigment, PBPs are safer and more commercially valuable than synthetic colorants. Synthetic colors have been shown or suspected to increase the risk of cancer and allergic reactions, have begun to uses of artificial colorants in foods have been restricted by many organizations in the world [4]. In contrast, the Food and Drug Administration (FDA) has approved PBPs use in food and cosmetic industries [5]. PBPs are strongly fluorescent and can thus be developed for novel immunofluorescent probing [6]. Meanwhile, PBPs have strong anti-inflammatory ability, for example, PC could act on NF-κB, TLR, PI3K/Akt/mTRO, and Nrf2 to inhibit inflammation [7]. In addition, PBPs have potential pharmacological activities in neuroprotection and strong inhibitory effects on the proliferation of cancer cells in lung, liver, and breast [8]. Meaningfully, these properties of PBPs show good application potential in the treatment of injuries and sequelae caused by COVID-19. Therefore, PBPs are expected from larger and broader market demands in these areas in foreseeable future.

**Fig. 1:**
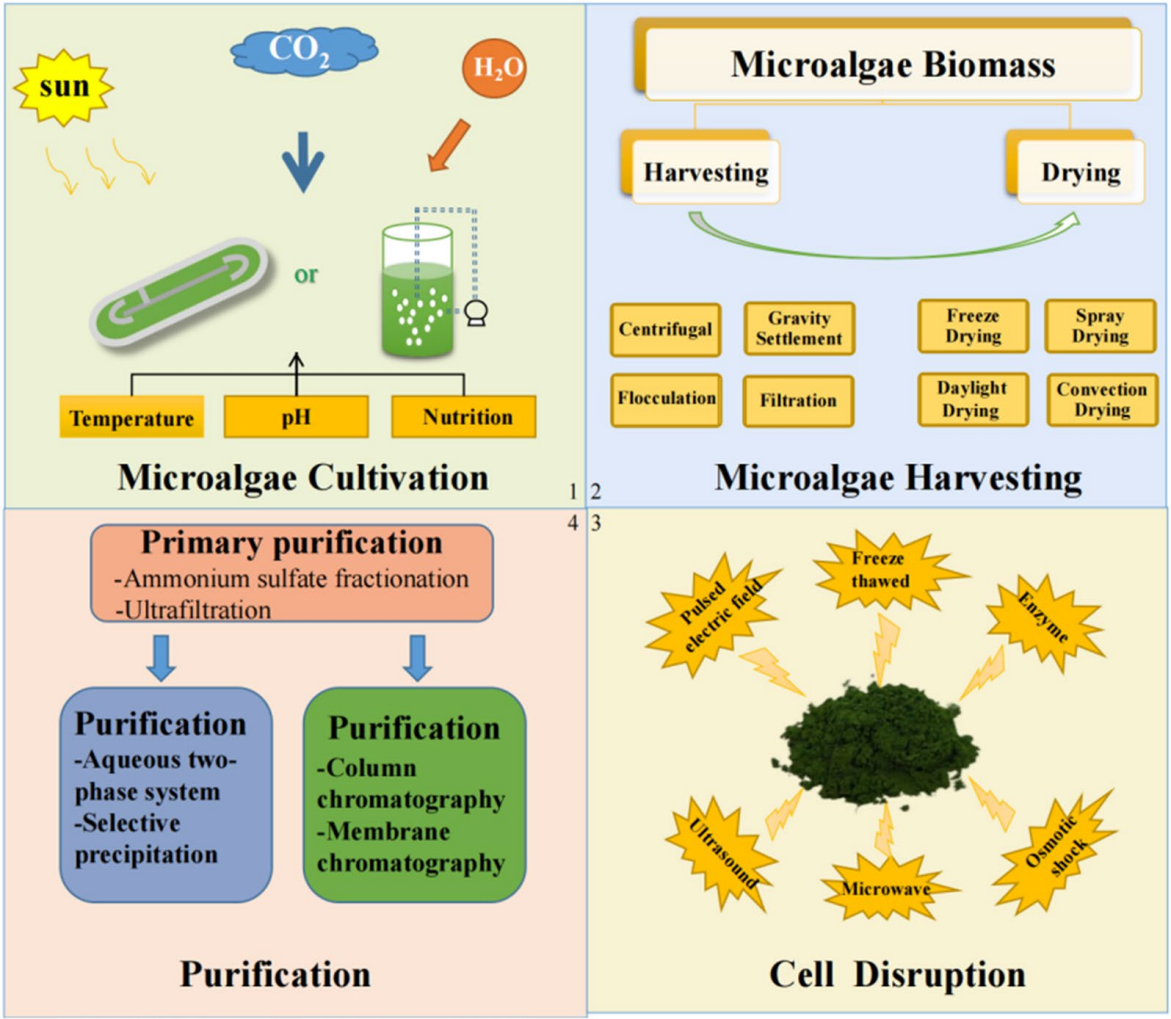
Steps of phycobiliproteins production

However, at present, the large-scale production of PBPs needs to be further optimized to meet the growing market demand. The production of PBPs includes upstream and downstream steps. The upstream contains microalgae cultivation, microalgae recovery, and desiccation, and the downstream includes cell fragmentation, and PBPs separation and purification. To achieve a large amount of PBPs accumulation in the upstream stage, improve the efficiency of downstream separation, purification, and continuous production capacity while reducing the production costs and pollution, which are challenging issues that must be solved for large-scale industrial production. In this review, approaches in the research into PBPs production technology are summarized (Fig. 1), the advantages and disadvantages of various technologies are commented, the potential of scale-up production is discussed, and the challenges and future development are prospected.

## Upstream of PBPs production

The upstream of PBPs production includes steps of biomass accumulation, biomass recovery, and dehydration. First, to produce PBPs, it is necessary to identify microalgal strains with high production to increase the growth through cultivation methods that conform to their growth characteristics, and then, to choose a cost-effective and best harvesting and drying methods for optimal recovery of PBPs biomass.

### Culture of microalgae and PBPs accumulation

The type and content of PBPs in different microalgae species vary in relation to the type of algal strain, bioreactor, and culture parameters. Choosing suitable algal strains and culture conditions can increase the production of target PBPs.

#### High-yield microalgae strain of PBPs

Different algal strains have different growth habits and biomass yields. Therefore, it is particularly important to choose suitable microalgal strains for large-scale production of PBPs.

##### Selection and breeding of high-yield microalgae strain of PBPs

Many microalgal strains including *Arthrospira platensis, Porphyridium* sp., and *Aphanizomenon gracile* are capable of producing PBPs (Table 1), [9, 10]. The isolation and selection of effective algal strains are key to the high productivity of PBPs. The first step in isolation is to collect samples from different ecosystems, e.g., rivers, lakes, etc., and new strains of microalgae are selected by comprehensive coordination among carbon and nitrogen sources, trace elements, pH, and temperature etc. [11], and then the PBPs productivity in algal strains can be enhanced by screening after isolation. Commonly, a large amount of algal strain is placed under the same conditions and finally the best strain is selected in terms of the production ability and the simplicity of procedures in culture. *Arthrospira* is widely used for production of PC for its easy availability and good growth performance, and has demonstrated a great market potential [12]. *“Spirulina”* is the commercial name for “*Arthrospira”*, and in scientific research, it is usually represented by “*Spirulina* (*Arthrospira*)” [13, 14]. Meanwhile, microalgae strains with high adaptability will have greater potential for application, As discovered by Limrujiwat et al. [15], *Nostoc* sp. SW02 algal strain has a high yield of PBPs (31.9%) in harsh production environments with insufficient light and nutrition, which is expected to become a new source of PBPs production.

**Table 1:** Microalgae strains containing PBPs

Strain type	PBPs	References
*Arthrospira platensis*	PC	[16]
*Synechococcus*	PC	[17]
*Arthrospira maxima*	PC	[18]
*Porphyridium purpureum*	PE	[19]
*Aphanizomenon gracile*	PC	[20]
*Rhodomonas salina*	PE	[21]

##### Transformation of high yield PBPs microalgae strains

Reasonable development and transformation of discovered microalgae strains is an important mean of improving the productivity of microalgae strains. Nowadays, genetic engineering is widely used to improve the growth ability, stress resistance, and other performance of microorganisms [22]. By modifying directionally the gene of microalgae strain, we can achieve the effect of high-yield PBPs. However, its transformant is instable and the efficiency of foreign gene expression remains low. Random mutagenesis is another way to produce different microbial mutants. Through chemical and physical methods, the genetic material of microalgae strains is induced to mutate, so that they can have new characters and inherit it stably, and then the mutants with high yield of PBPs could be screened out for cultivation. In a study, *Arthrospira platensis* was mutated with G n-methyl-n-nitro-nitroguanidine (NTG), and three morphological mutants (G-1, G-2, and SF) were selected and identified. Compared with the wild type, G-1 and G-2 showed higher biomass and PC content. These two mutants have the potential for commercial production of PC [22].

#### Bioreactors

Bioreactors are another important factor on algal culture. There are two types of bioreactors for microalgae culture: open bioreactors and closed bioreactors. Table 2 describes the size and production capacity of several different reactors.

**Table 2:** Bioreactors used to produce PBPs

Reactor type	Microalgae species	PBPs	Scale (dm^3^)	Remarks	References
Outdoor raceway ponds	*Arthrospira platensis*	PC	21.5	PC productivity 8.5 mg/(L·d)	[23]
Thin-layer raceway ponds	*Nostoc calcicola*	PC	80	PC content about 12 mg DW/g	[24]
Flat-plate photobioreactor	*Arthrospira platensis*	PC	3	the maximum concentration of PC 67.54 mg/(L^­1^)	[25]
Annular photobioreactor	*Arthrospira platensis*	PC	6	PC productivity 80 mg/(L·d)	[26]
Continuous photobioreactor	*Porphyridium purpureum*	PE	0.7	PE productivity 16.93 mg/(L·d)	[27]
Twin-layer porous substrate Bioreactor	*Galdieria sulphuraria*	PC APC		PC productivity 0.28 g/(m^2^·d) APC productivity 0.23 g/(m^2^·d)	[28]

##### Open ponds

Open ponds are suitable for large-scale culture of microalgae, and are less expensive than closed systems. Open ponds include natural lagoons, raceway-type ponds, etc., and was used initially for expanding microalgae culture. Among them, raceway-type ponds are the most common one, which usually consist of an array of flumes of equal length and width at depth of 0.2–0.4 m, in which water is circulated by mechanical paddle wheels to prevent cell settling [29]. Open ponds are often exposed to contamination. Yu et al. [30] tested the use of microfiltration membrane for filtering algal media in continuous culture of *Arthrospira platensis* microalgae in open ponds. Their results show that the microfiltration medium could improve biomass production, photosynthesis, and PC accumulation, and reduce microbial contamination during non-stop cultivation. To increase the light-harvesting efficiency in open bioreactor, Raeisossadati et al. [23] investigated a luminous solar concentrator (LSC) for increasing light delivery to water bottom of an outdoor pond under a red LSC, by which the production of *Arthrospira platensis* PC [8.5 mg/(L·d)] was increased by 44% from that of the control. Open ponds are easy to clean and maintain, and have direct sunlight and low dissolved oxygen accumulation. However, biomass production in open pond was easily affected by climate, with low CO_2_ fixation efficiency and high water evaporation [31], which limited greatly the application in many countries of the world [32].

##### Photobioreactors

The use of photobioreactor is common within laboratories research, including the following types: tubular or flat, inclined or spiral, and biofilm bioreactors (Table 2). Enclosed reactors are usually small, but they can be designed to suite algal growth requirements by manipulating culture conditions in light, acidity, and temperature, which promoted the accumulation in biomass of PBPs [33]. Sun et al. [34] addressed the light-dead zone problem by embedding hollow polymethylmethacrylate (PMMA) tubes in a flat plate photobioreactor to increase the light in the light-harvesting dead zone, which improved the efficiency and increased the biomass yield by 23.42%, this design has the potential to be applied to the production of PBPs in photobioreactors. Additional energy input is usually required to maintain optimal temperature in a photobioreactor, Nwoba et al. [32] used an insulated photobioreactor that was integrated with photovoltaic panels for the cultivation of *Arthrospira platensis,* biomass productivity, and corresponding C-PC content that are, respectively, 67% and 45% higher than those achieved in a classical raceway that was heated continuously. This design presents a less expensive and more energy-efficient pathway to large-scale microalgal culture. The photobioreactor avoided the exchange of substances with outside environment and reduced the risk of contamination. However, the production cost was still high. Only a small amount of photobioreactor can be used for commercial scale cultivation of microalgae to produce PBPs.

#### Nutrition mode

Microalgae produce PBPs through photosynthetic autotrophic, heterotrophic, or mixed nutrient modes. Photosynthetic autotrophy is often used for PBPs production in open-pond culture for microalgae, in which inorganic carbon (CO_2_) is reduced into carbohydrates via photosynthesis for algal growth and development [35]. Heterotrophic production is not limited by light, and the extreme growth environment reduces the risk of contamination. [36]. *G. sulphuraria* is an alga that can grow at very low pH, capable of growing heterotrophically and produce PC using glucose, fructose, sucrose, etc., and the PC yields could reach up to 30 mg/g in batch replenishment and high-density continuous culture [37]. It was also shown that the specific growth rate of mixed nutrient cultures was much higher than that of photoautotrophic or heterotrophic nutrients during the synthesis of PBPs [38]. Morais et al. [39] evaluated the effect of mixed nutrition and heterotrophy of *Aphanothece microscopica* Nägeli on PC production. They found that after 12 h incubation, the PC yield in mixed nutrition was higher (1.50 mg/g) than that of heterotrophic culture (1.39 mg/g). Chemical energy from light-sourced carbon and organic carbon increased the biomass productivity, making the mixed-nutrition culture mode more suitable for large-scale production [40].

#### Factors affecting cultivation

The ability to produce PBPs from algal biomass depends closely on various environmental factors on algal growth and PBPs accumulation (Table 3). For example, light, temperature, CO_2_, and nutrients can significantly affect algal biomass accumulation [41].

**Table 3:** Effects of different culture factors on the accumulation of PBPs in microalgae

Influencing factors	Microalgae species	PBPs	Basic culture condition	Remarks	References
Light intensity	*Nostoc sphaeroides* Kützing	PC	25 °C;	PC contents were the highest under white light at 90 μmol m^­−2^ s^­−1^ or blue light at 90 μmol m^­−2^ s^­−1^	[42]
Different ratios of nitrogen to phosphorus	*Microcystis aeruginosa* (FACHB-905)	PC	25 °C; 12:12 h light:dark cycle; 40 μmol photons m^­−2^ s^­−1^	PC contents were the highest (8.3 mg·L^−1^) under NO_3_/P of 30 ~ 50	[43]
Different nitrogen sources	*Phormidium* sp. EGEMACC72	PC	22 °C: 80 μmol photons m^­−2^ s^­−1^	PC contents were the highest (19.38 ± 0.09 mg·L^­-1^) when ammonium chloride was used	[44]
Nitrogen concentration	*Spirulina (Arthrospira) platensis*	C-PC	28 °C: 100 μmol photons m^­−2^ s^­−1^	C-PC increased with nitrogen (0.03–0.045 M)	[45]
Temperature	*Arthronema africanum*	C-PC	150 μmol photons m^­−2^ s^­−1^	C-PC amounting to 23% of the dry algal biomass at 36 °C	[46]
Metal ions	*Arthrospira platensis*	PC APC	32 °C; 12:12 h light:dark cycle; white fluorescent tubes (35 μEm­^−^^­2^ s^−1^)	The highest PBPs productivity under ferrous sulfate at a concentration of 0.1 g·L^−1^	[47]
Temperature	*Biflagellate microalga*	PE	White light; 12:12 h light:dark cycle; 15 μmol photons·m^­−2^ s^­−1^	The highest PE productivity (1.594 μg·mL^­−1^·d^­−1^) at 26 °C	[48]
Different carbon supply	*Porphyridium purpureum*	B-PE	26 °C; 10, 000 lx	The maximum B-PE content was 12.17% when 2 g·L^­−1^ NaHCO_3_ was added	[49]

##### Light

For microalgal species, light is a vital factor on their growth and survival. Microalgae have different adaptive strategies in their bodies to light and accumulation of PBPs varies under different light conditions [48]. Studies have shown that different wavelengths can enhance the production of specific compounds. Ma et al. [42] found that blue light at 30 μmol m^­−2^·s^­−1^ or white light at 90 μmol m^­−2^·s^­−1^ was most beneficial for growth and accumulation of PBPs in *N. sphaeroides*. There was also an effect of light intensity on algal plant growth and PBPs synthesis [50]. Xie et al. [21] found that the PE content of *Rhodomonas salina* increased with time at low light intensities (20 and 100 μmol m^­−2^·s^­−1^) and decreased with time at high light intensities (150 and 250 μmol/(m^2^·s)) the PE content decreased with time. Second, although microalgae are phototrophic organisms, they needs to be given a corresponding amount of dark time during the culture. Light–dark cyclic culture facilitates algal growth and accumulation of PBPs [12]. Ho et al. [51] found that using white LED as the light source coupled with optimal light–dark frequency (30 min:30 min), recycled medium (50% replacement), and nitrate addition (45 mM), the highest C-PC content and productivity of 14.9% and 101.1 mg/L/d, respectively, were achieved, and reduced energy consumption by 10–45% in a culture of *Spirulina* (*Arthrospira*) *platensis* for C-PC production.

##### Nitrogen source

Microalgae are able to use various nitrogen sources, e.g., ammonium NH^4+^, nitrate, nitrite, urea, etc. However, the ability is different among algal strains. Khazi et al. [44] compared the effects of using nitrate (NaNO_3_ and KNO_3_) and ammonium (NH_4_Cl) in different algal strains. Among them, *Arthrospira platensis* showed the highest total PBPs accumulation with NaNO_3_ supplementation (22.27% ± 0.2%, dry weight) and *Pseudoscillatoria* sp. had the highest PBPs under NH_4_Cl supplemented culture conditions (19.99% ± 0.14%, dry weight). In addition, the amount of nitrogen source addition was not proportional to the final PBPs production, and high nitrogen concentration may limit the growth of microalgae [52]. Chen et al. [45] found that the PC content in *Spirulina* (*Arthrospira*) *platensis* increased with the increase of nitrogen concentration (0.03–0.05 M), and when nitrogen concentration increased to 0.09 M, no significant change took place in PC production. Therefore, appropriate addition of nitrogen source is conductive for producing more PBPs.

##### Temperature

Temperature affects the production of PBPs. Ideal conditions for growth and metabolite production depend on temperature adaptation of a specific strain. *Cyanobacterium Arthronema africanum* has a reduced content of PBPs at a low (< 15 °C) or high temperature (> 47 °C) [46]. Park et al. [53] reported a wide range of suitable temperature at 10–35 °C for good growth performance of *Arthrospira maxima.*

##### Others

Microalgal cultures require a variety of nutrients and chemical elements, such as carbon, nitrogen, iron, potassium, calcium, copper, cobalt, iron, zinc, and manganese. Inappropriate supply of these element would affect the accumulation of PBPs. Usually, the production of a specific product can be increased by optimizing the concentration of each component of the culture medium. Zuorro et al. [54] investigated the effects of NaNO_3_, Na_2_CO_3_, K_2_HPO_4_, and trace metal concentrations in BG-11 medium on the production of PBPs by *Oscillatoria* sp. After optimizing the concentration ratios, the C-PC, APC, and PE contents increased 2.12-fold, 1.77-fold, and 4.17-fold, respectively. Shashirekha et al. [55] studied the role of Cr^3+^ in several cyanobacteria, they added Cr^3+^ to the medium of *Lyngbya* sp. and *Oscillatoria* sp. and found that it was able to increase the production of PBPs. On the contrary, the production of PBPs was reduced when Cr^3+^ was added to the media of *Synechocystis* sp., *Aulosira* sp., and *Nostoc* sp. [55]. pH affects the accumulation of PBPs by affecting thew physiochemical functions. In addition, the production of PBPs in algal plants can be enhanced by adding growth hormones (GA3, IBA, and IPA) to the culture to promote the use of nutrients by the microalgae and thus enhance the production of PBPs.

### Collection of microalgae biomass

After microalgae cultivation is completed, additional steps are required to convert the biomass into a feedstock for PBPs production. Isolation and concentration of biomass from algal commonly uses methods include centrifugation, flotation, flocculation, and membrane filtration etc. [56, 57] (Table 4).

**Table 4:** Harvesting methods of microalgae biomass

Recovery methods	Microalgae species	Control conditions	Remarks	References
Centrifugation	*Synechococcus elongatus* PCC 7942	Speed, time	High efficiency, no pollution; high energy consumption	[58]
Ultrafiltration	*Cyanobacteria Syn7942, Syn6803, and Ana7120*	Aperture size, pressure	Large amount of processing, easy to enlarge; membrane life	[59]
Electrocoagulation–flotation (ECF)	*Spirulina (Arthrospira) platensis*	Electrode type, current intensity	Low energy consumption, high efficiency; may cause pollution	[60]
Compound buoyant-bead flotation	*Spirulina (Arthrospira) platensis*	Time, pH	High efficiency	[61]
Flocculation	*Spirulina (Arthrospira) platensis*	Type of coagulants, amount added	High efficiency; may cause pollution	[62]
Autoflocculating	*Arthrospira platensis*	Type of microalgae strains	No pollution	[63]

#### Traditional physical methods

Traditional physical methods include gravity settling and mechanical recovery to avoid the impact of chemicals on PBPs. Centrifugation is one of the most commonly used methods. Relying on the generation of a centrifugal force which acts radially and accelerates the movement and separation of particles based on the difference in density between the particle and the medium surrounding it, and biomass can be recovered in a short time. However, the energy consumption and instrument cost are high and the processing amount is insufficient, which has been used mostly in small-scale application [64]. Gravity sedimentation recovery of microalgae uses gravity as agent, has low requirements on external conditions, and can be easily applied to outdoor ponds. Although the recovery efficiency is low, it is more cost-effective [65]. Used in conjunction with flocculants, gravity method is potential to be used in microalgae mass culture. Another collection method is to use membrane filtration technology at low energy consumption. Membrane technology has the advantages of simple operation and easy scale-up, which is a more economical way to harvest [66], and plays a great role in dewatering and harvesting algae [67]. The membrane filtration efficiency is affected by various conditions, such as membrane properties, hydrodynamic conditions, and suspension characteristics, while membrane contamination such as biofilm buildup and fouling are major challenges for applications in microalgae recovery [68].

#### Flocculation

Flocculation methods are classified as physical flocculation, chemical flocculation, and biological flocculation [69]. Electrolysis is a commonly used physical flocculation method to collect algal biomass through physical reactions triggered by different electrodes. Physical flocculation performs well in laboratory or pilot factory scale, but it needs special devices, thus the economic return is low, which limited the bulk production [70]. Chemical flocculation is a method in which inorganic flocculants such as aluminum salts or iron salts is applied to collect microalgae. Organic flocculants such as chitosan and surfactants can also promote the flocculation by changing the solubility and electronegativity of biomass. Labeeuw et al. [71] uesd polyacrylamide as a flocculant to flocculate microalgae, and achieved above 82% efficiency in pilot scale. However, the flocculants have a certain impact on the integrity of cell membranes. The additional cost and possible pollution are major drawbacks in the use of chemical flocculation. Hansel et al. [72] found cationic starch could flocculate microalgae at low doses without causing pollution, thus it is a potential microalgae flocculant and has the feasibility of commercially recovering algae biomass. Compared to chemical flocculation, biological flocculation is considered more eco-friendly. Biological flocculation can either be in self-flocculation or be the result of a combination of multiple factors [73]. This method is more eco-friendly and economical, but it is used on a laboratory scale only. At present, no successful large-scale microalgae cultivation in this method has been reported [70].

### Drying of microalgae biomass

In addition to harvesting, the selection of the most suitable drying method should also be considered to avoid the extreme operating environment that may affect the activity of PBPs for high-quality biomass and to reduce production costs. The dehydration methods include spray drying, freeze drying, convection drying, and sunlight drying [74] (Table 5).

**Table 5:** Drying methods of microalgae biomass

Methods	Classification	Advantage	Disadvantage	References
Solar drying		Low processing cost; No equipment required	Weather dependent, may cause pollution	[75]
Centrifugation dewatering	Bucket centrifuge	Efficient	High energy consumption; small processing capacity	[76]
Spray drying	Centrifugal spray drying; pressure spray drying; airflow spray drying	Rapid and efficient drying	High operating cost	[77]
Freeze drying	Air freeze drying; vacuum freeze drying	No pollution; convenience	High operating cost	[78]
Convective drying	Belt drying; oven drying; tray drying; tunnel drying	Efficient for large scale processing	High temperature may destroy protein	[79]

Most of the energy in solar drying comes from the sunlight and it is the least-cost method of drying microalgae. On the other hand, this also means that solar drying depends on weather conditions and is only suitable for the areas with ample sunlight. Meanwhile, exposure to open environments may increases the risk of pollution. Spray drying has great advantages of rapid dehydration for protecting active components biologically; however, it may lose volatile components and thus not suitable to produce of heat-sensitive substances [80]. Freeze-drying does not affect significantly the cell structure, but the cost is high and drying time is long, which also limit its application in a large-scale production. Convective drying is also one of popular methods of drying microalgae biomass. By tuning the reaction temperature, the biomass can be well-dried. Seghiri et al. [81] compared the effects of freeze drying, spray drying, and convection drying on the extraction of PBPs from *Arthrospira platensis*. They noticed that the three drying methods had no significant difference in total protein, but significant difference in the content of water-soluble protein. In terms of yield, convection drying had the highest PBPs yield (6.215%), followed by freeze drying (4.629%), and spray drying (4.382%). Although convective drying is the highest among the three methods, the efficiency is still low, the application in large-scale production needs more research, and the efficiency shall be heightened and energy consumption reduced.

## Downstream of PBPs production

The downstream stage is an important stage related to the purity and yield of PBPs, including cell disruption and separation–purification. Through the combination of different technologies, high-purity PBPs can be obtained (Table 6). The amount of PBPs in the sample was usually calculated using simultaneous equations of Bennett and Bogorad [82] and the extinction coefficients from Bryant et al. [83]. The purity of PBPs is represented by the ration of PBPs content to the total protein content, which is usually expressed by the ratio of A_λmax_ nm/A_280_ nm.

**Table 6:** Production of PBPs

PBPs	Microalgae species	Cell disruption + crude extraction	Purification	Purity	Yield (%)	References
PC	*Spirulina *(*Arthrospira*) *platensis*	High pressure homogenization	ATPS + anion-exchange chromatography	6.69		[84]
PC	*Spirulina *(*Arthrospira*) *platensis*	Homogenized	Aqueous two-phase extraction	4.32	79	[85]
PC	*Spirulina *(*Arthrospira*) *platensis*	Freezing and thawing + ammonium sulfate fractionation	Anion-exchange chromatography	4.5	14	[86]
PC	*Limnotrhrix* sp.	Freezing and thawing + ammonium sulfate fractionation	Activated charcoal and chitosan + tangential cross-flow filtration	4.3	8	[87]
PC	*Phormidium fragile*	Grinding under liquid nitrogen + ammonium sulfate fractionation	Hydrophobic interaction chromatography	4.52		[88]
PC	*Arthronema*	Freeze–thawed	Rivanol treatment	4.52	55	[89]
APC				2.41	35	
PC	*Spirulina fusiformi*	Freezing and thawing	Rivanol treatment	4.3	46	[90]
PC	*Aphanizomenon flos-aquae*	Ammonium sulfate fractionation	Hydroxyapatite chromatography	4.78		[91]
PC	*Spirulina *(*Arthrospira*) *platensis*	Homogenized	Anion-exchange chromatography	3.43	77.3	[92]
PC	*Synechocystis aquatilis*	Osmotic shock	Expanded bed chromatography + anion-exchange chromatography	> 4.0		[93]
PC	*Calothrix* sp.	EDTA and lysozyme treatment	Anion chromatography + hydrophobic interaction chromatography	3.5		[94]
PC	*Euhalothece* sp.	Freezing and thawing + ammonium sulfate fractionation	Activated carbon adsorption + UF	5	60	[95]
PC	*Anabaena fertilissima* UPCCC	Freezing and thawing + ammonium sulfate fractionation	Ion exchange chromatography + size exclusion chromatography	3.28	28	[96]
PC	*Synechococcu* sp.	Ultrasound + ammonium sulfate fractionation	Ion-exchange (DEAE-cellulose) chromatography	4.03		[97]
PC	*Spirulina *(*Arthrospira*) *platensis*	Ultrasound + ammonium sulfate fractionation	Gel-filtration column	3.5		[98]
PC	*Arthrospira platensis*	Osmotic shock	Activated charcoal + Sephadex G 100 + DEAE sepharose fast Flow	3.25	48.2	[99]
PC	*Arthrospira platensis*	Ultrasound	Activated carbon adsorption	1.23	80	[100]
PC	*Spirulina *(*Arthrospira*) *platensis*	Milling + ammonium sulfate fractionation	Ion exchange chromatography	4.0		[101]
PC	*Arthrospira platensis*	Microwave	Ionic liquids	1.22		[102]
PE				0.71		
APC				1.03		
PC	*Spirulina* (*Arthrospira*) *platensis*	Freezing and thawing	ATPS	2.38		[103]
PC	*Spirulina* (*Arthrospira*) *platensis*	Freezing and thawing	ATPS + UF	2.11		[104]
C-PC	*Spirulina* (*Arthrospira*)* platensis*	Ultrasound	Liquid biphasic flotation technique	3.49	90.4	[105]
PC	*Arthrospira platensis*	Ultrasound	ATPS + dialysis + precipitation	4.22		[106]

### Cell disruption

PBPs are a water-soluble intracellular protein. Selecting appropriate conditions for cells to release PBPs into the buffer, and then separate, purify, and obtain natural PBPs, while their original structure and function remain intact, which is the extraction steps of the whole PBPs and one of the most critical steps in the purification process. When breaking cells, one should avoid a violent method that may destruct the structure and properties of PBPs. In addition, violent methods may dissolve out a large number of impurities from cells, such as polysaccharides, which makes the separation harder. Cell disruption techniques usually include non-mechanical methods (i.e., repeated freezing and thawing, osmotic shock, chemical reagents, and enzymatic methods) and mechanical methods (i.e., high-pressure homogenization, bead milling, ultrasound, and microwaves).

#### Non-mechanical methods

Non-mechanical methods are used to break cell walls at a low temperature or by adding specific chemicals. Freezing-and-thawing is one of the common methods of cell-wall breaking in the laboratory. Microalgae are frozen at − 20 °C and then thawed at 4 °C to break cell wall and release intracellular substances [107]. Repeated freezing-and-thawing can often obtain greater efficient of cell-wall breaking. Osmotic shock is a simple extraction method by which PBPs can be released by variation in internal or external osmotic pressure after microalgae are mixed with distilled water, or placed in extraction buffer away from light for several hours. This method is easy to scale up for a large application. On the other hand, by adding chemical reagents, such as surfactants or organic solvents into the sample solution, cell walls can be broken up, or by adjusting the pH value of the buffer, the electron-bearing properties of protein can be altered thus to increase the solubility of the product and release the intracellular substances. This method is efficient, but has a risk of contaminating the sample [108]. Alternatively, enzymatic hydrolysis of cell walls is a greener and milder method, but the cost is relatively high, which is not conducive to a commercial use.

#### Mechanical methods

Mechanical methods use physical force to disrupt cells. High-pressure treatment crushes microalgae through high shear and pressure generated by machine for PBPs. This method features uniform treatment and produces little heat, but attention should be paid to the pressure tolerance by different microalgae. Ultrasonic assisted extraction (UAE) is a method of destroying microalgae cell walls using low-frequency ultrasound to generate a large amount of heat and shock waves through a liquid medium. The higher the ultrasonic frequency, the greater the crushing effect. Microwave-assisted extraction can quickly extract PBPs, but as the time increases, thermal denaturation of PBPs may occur [102]. Pulsed electric field (PEF) is an emerging physical extraction method characteristic of non-thermal, less energy cost, and environmentally friendly. Pulse discharging could cause cell membrane electroporation or cell membrane electro-disintegration, and promote the destruction of cell homeostasis structure, and thus release PBPs [109]. Martínez et al. [110] evaluated the application of PEF in *Arthrospira platensis* treatment, in which higher extraction rates was obtained by adjusting the electric field intensity, temperature, and treatment time, but they found that a period of time was needed after PEF treatment for better extraction of PBPs. However, the longer time requirement may limit large-scale production [111]. Using plasma generated by high-voltage discharge to break cell wall is also an efficient method, which includes spark discharge and corona discharge. The spark discharge promotes protein extraction by disrupting the cell wall via several ways, including strong shock waves, electric fields, UV radiation, and generation of reactive substances [112]. The spark discharge treatment of algal suspensions has been shown an effective and mild way to microalgal disintegration as well as pigment and protein extraction [113]. Sommer et al. [113] compared spark discharge, pulsed electric field, and ultrasound treatment of *Cyanidium caldarium* for 30 min for PC extraction; they showed that the ultrasound and spark discharge had higher extraction efficiency, and the PC purity was the highest in the original extract of spark discharge. Although mechanical breakage methods have the advantages of high efficiency and zero pollution, they often require special device, and insufficient processing capacity, high installation cost, and energy consumption limit their application in large-scale production.

Due to different structures of microalgae cells, it is usually difficult to achieve good wall-breaking effect by a single method. Therefore, different methods are often combined to increase the efficiency of wall-breaking, such as ultrasound-assisted and enzymatic combination, microwave and enzymatic combination, and ultrasound and freeze–thaw combination, etc., which have achieved better extraction results.

### Preliminary extraction of PBPs

After cell disruption and solid–liquid separation and deslagging, PBPs are usually preliminary extracted and purified using ammonium sulfate fractionation or ultrafiltration adsorption according to the differences in molecular weight, solubility, and charge ion properties of PBPs. Ammonium sulfate precipitation is one of the most widely used methods. By continuously increasing the content of ammonium sulfate in the solution, the stability of the surface colloid of PBP is destabilized, which makes them precipitated out in batch, and then the solid precipitates containing different types of PBPs can be obtained by centrifugation. Ultrafiltration is a technique that used to separate natural sensitive compounds by a combination of membrane pore blocking, membrane surface mechanical sieving, and membrane pore adsorption, which can separate PBPs in higher molecular weight by ultrafiltration. As the treatment process is relatively mild, the configuration, conformation, and optical activity of PBPs do not alter in general. Nisticò et al. [114] used 20 kDa polyethersulfone (PES) membranes to ultrafiltrate crude extracts of *Arthrospira maxima* from water, and the ultrafiltration removed about 91.7% of DNA, thus improved the purity of PC in the material retained. The ultrafiltration method can achieve rapid separation of crude extracts of PBPs on large scale with simple procedure and has a large potential for application.

### Purification of PBPs

Purification of PBPs is an important step in the production of high-purity PBPs. Typically, after purification by chromatographic chromatography and aqueous phase extraction, the purity of PBPs can reach reaction grade, analytical grade, or above, which could greatly improve the value of PBPs.

#### Chromatographic technology

Various chromatographic techniques have been used to purify PBPs [115]. Gel filtration chromatography, hydrophobic exchange chromatography, and ion exchange chromatography are common laboratory methods. Soni et al. [88] treated *Phormidium fragile* samples by liquid nitrogen grinding with ammonium sulfate precipitation, followed by hydrophobic chromatography, sulfate precipitation, after which PC in purity 4.42 was obtained. Patel et al. [116] used DEAE ion exchange chromatography to purify Spirulina (Arthrospira) sp. samples via freeze-thaw with ammonium sulfate precipitation, after which PC in purity 4.42 was obtained. Moreover, expanded bed adsorption chromatography is a faster technique that reduces the number of operation for protein adsorption and separates purified proteins directly from crude extracts [117]. Higher purity PBPs can usually be obtained by column chromatographic techniques, but the cost and yield could not meet the commercial demands. Membrane chromatography is considered an effective alternative to the column chromatographic techniques, it utilizes natural or synthetic membranes with selective permeability, allowing easier separation and purification of large molecule proteins. The separation of membrane chromatography simply combines the ability of chromatography and the speed of microfiltration, and can be used to purify various commercially valuable PBPs [118]. Ng et al.[119] prepared a chitosan-modified nanofiber membrane and quickly purified *Arthrospira platensis* C-PC in negative chromatography; and the results show that the selectivity of membrane for protein was in the order of troublesome contaminating proteins (TP) > APC > C-PC, because C-PC molecules could easily penetrate into the membrane without being adsorbed, thus improving the purity. Zang et al. [120] used PVDF membrane to purify crude extract of *Pyropia yezoensis* precipitated by ammonium sulfate in 5–10 min, and obtained R-PE in purity of 4.25, which greatly improved the separation and purification efficiency of PBPs*.* This also seems to be applicable for the purification of PBPs in microalgae. Chaiklahan et al. [121] used a 50 kDa membrane to purify the crude extract of *Spirulina* (*Arthrospira*) sp*.*, and obtained a PC in purity of 1.07, which does not need to add other substances, and showed a good prospect in mass production of food-grade PC. At the same time, with the improvement of the membrane, its service life is enhanced and its separation efficiency is increased, which has the potential to be used in large-scale production.

#### Aqueous phase extraction technology

Aqueous two-phase system (ATPS) is an efficient liquid–liquid extraction technique of purifying proteins. It uses two immiscible mixed polymers, or a polymer and a salt to separate molecular under intermolecular forces. ATPS features high extraction rate and scalability and seems to be a good candidate to replace traditional methods [122]. Zhao et al. [104] used ATPS to purify *Spirulina obtusifolia* with PEG-1000 and NaNO_3_, and obtained C-PC in purity of 2.11 from the top distribution item. Phong et al. [123] added an air flotation system to ATPS, which could adsorb surface active compounds of biomolecules at the surface of rising bubbles, and bring the active compounds from the bottom aqueous phase to the top organic phase, which accelerated the system process, obtained higher purity PBPs, and was able to scale up the production. In addition, ATPS can be combined with an ultrafiltration system to remove polymers from the product and increase the concentration of the extract.

#### Other methods

To improve the capacity for large-scale production of PBPs and increase economic benefits, various new separation and purification technologies have been developed. Huang et al. [124] established three-phase partitioning process (TPP) to extract and separate simultaneously phycobiliproteins and polyunsaturated fatty acids (PUFA) of wet *Porphyridium* biomass. Under optimized conditions, the extraction recovery yield of B-PE, APC, PUFA, and other substances in the TPP process exceeded 90%. Similar results were achieved in pilot scale (20 L). According to Martins et al. [125], by adding NaPA8000 to the crude extract of *Gracilaria gracilis*, R-PE was selectively precipitated, and then ultrafiltrated, from which high purity R-PE (87.3%) was obtained. However, whether this method can be applied to the extraction of microalgae PBPs remains poorly studied. Lauceri et al. [126] established an innovative ultrasound-assisted cell lysis process to extract PC from *Arthrospira platensis,* using ammonium sulfate to reduce the release of PBPs in the solution during the ultrasound-assisted lysis purification. Followed by extraction using H_2_O, CaCl_2_, or NaCl, a PC product in purity of 2.5–3.5 was obtained, which greatly reduced the purification steps and time.

## Challenges and prospects

PBPs are an important biological resource in food, cosmetic, and biomedical industries. Therefore, large-scale PBPs production is necessary, for which the selection of high-yield algal strains is a prerequisite to the yield of PBPs. According to the growth habit and production scale requirements of the algal strains, an appropriate reactor shall be selected considering the manipulation of conditions required by the reactor for large efficiency. The mixotrophic approach is undoubtedly the most efficient way of culture to maximize the accumulation of PBPs. Making use of industrial wastewater and seawater as nutrient sources is a green approach to achieve multifaceted use of resources while reducing the cost. However, excessive salts and pollutants in wastewater may affect algal growth [127]. Using physical machinery for algal biomass recovery is expensive, while using chemical flocculation to recover biomass demonstrated greater advantages. In the near future, inexpensive flocculants will be formulated for commercial use. Dewatering and drying should be paid with close attention to the possible loss of PBPs due to the extreme conditions of treatment and also to the balance between energy consumption and efficiency.

The downstream part of the production of PBPs controls the quality. As PBPs are heat-sensitive substances, high temperature must be avoided to prevent them from decomposition. The cell disruption and purification depend on the balance between production cost and production capacity. New technologies using microwave, ultrasound, and pulsed electric field have high efficiency, but mostly are used at laboratory level and are difficult to meet the demands of large-scale continuous production. In contrast, pulsed spark discharge is a mild and efficient mean of cell wall breaking and has potential for application in large-scale production. Purification is normally done in hydrophobic chromatography, ion chromatography, etc., but it is time cost. Membrane chromatography is more rapid, easy to scale up, and thus has better prospect for application. ATPS needs no chromatography to complete the separation and purification. Ruiz-Ruiz et al. [128] designed an aqueous two-phase system coupled to a spiral continuous extractor achieved enhanced extraction of *Spirulina* (*Arthrospira*) *maxima* PC and increased the efficiency of separation. The separation solvent used in a continuous extractor shall be developed in the future to increase automation and improve the separation and purification proficiency. Although the yield and purity of PBPs are important factors, economic returns must be considered for large-scale production. In addition, an issue in laboratory scale may become a greater impact on industrial production. Pilot experiments are necessary before large-scale practice is put forward. At last, to realize industrial continuous, and automated production of PBPs is the future direction of development.

## Summery and conclusion

In this paper, the modern technology of extraction and purification of PBPs from microalgae culture is commented; the characteristics of different types of the technology from the selection and breeding of algal strains, bioreactors, to the selection of nutrition methods are introduced and commented; the influencing factors of culture, harvesting of algal biomass, drying, cell crushing, separation, and purification are reviewed; and the possibility of scaling-up production is discussed. At last, the future prospect and challenge in the field of industrial production of PBPs are viewed. It is the authors’ hope to provide an insightful review for the industrial production of PBPs.

## Data Availability

All data analyzed are included within this article.

## Abbreviations

PBPs: Phycobiliproteins
PC: Phycocyanin
PE: Phycoerythrin
APC: Allophycocyanin
PEC: Phycoerythrocyanin
FDA: The Food and Drug Administration
GA3: Gibberellic acid
IBA: Indole-3-butyric acid
IPA: Indolepro pionic acid
UAE: Ultrasonic assisted extraction
PEF: Pulsed electric field
PES: Polyethersulfone
UF: Ultrafiltration
DEAE: Diethylaminoethyl cellulose
TP: Troublesome contaminated protein
ATPS: Aqueous two-phase system
TPP: Three-phase partitioning
